# Clinical evaluation of pediatric olfactory disorders: a review from etiology to management

**DOI:** 10.3389/falgy.2026.1741382

**Published:** 2026-01-21

**Authors:** Eleonora M. C. Trecca, Marella Reale, Luca Leone, Michele Cassano, Antonio della Volpe, Ignazio La Mantia, Fabio Pagella, Elena Cantone

**Affiliations:** 1 Department of Otorhinolaryngology-Head and Neck Surgery, University Hospital of Foggia, Foggia, Italy; 2 Otorhinolaryngology Unit, Head and Neck Department, Meyer Children’s Hospital IRCCS, Florence, Italy; 3 Otolaryngology Department, Santobono-Posillipon Hospital, Naples, Italy; 4 Department of Medical and Surgical Sciences and Advanced Technologies “GF Ingrassia”, ENT Section, University of Catania, Catania, Italy; 5 Department of Surgical Science, University of Pavia, Pavia, Italy; 6 Department of Otorhinolaryngology, Fondazione IRCCS Policlinico San Matteo, Pavia, Italy; 7 Department of Pharmacy, Health and Nutrition Sciences, University of Calabria (UNICAL), Cosenza, Italy; 8 Otolaryngology Unit, Annunziata Hospital, Cosenza, Italy

**Keywords:** adenoid hypertrophy, hyposmia, Kallman syndrome, olfactory assessment, olfactory training, otolaryngology, pediatric olfactory disorder

## Abstract

Olfactory perception plays a fundamental role in nutrition, emotional development, and social behavior, yet olfactory disorders (OD) in children remain largely underrecognized and understudied. This mini review summarizes current evidence and proposes a structured clinical approach for the evaluation and management of pediatric OD. Etiologies are diverse, encompassing congenital syndromes such as Kallmann and CHARGE, post-infectious and post-traumatic forms, inflammatory airway diseases, and structural or iatrogenic causes. Accurate diagnosis begins with a detailed medical history and comprehensive ENT examination, complemented by psychophysical olfactory testing adapted for pediatric populations. Although several validated tools exist—such as the Sniffin’ Sticks, U-Sniff, Pediatric Smell Wheel, and pBOT-6—standardized age-specific protocols and normative data remain limited. Imaging techniques, particularly MRI, provide valuable insights into congenital and acquired abnormalities of the olfactory bulbs and tracts, while CT is reserved for sinonasal or bony pathology. Multidisciplinary collaboration among pediatricians, neurologists, endocrinologists, geneticists, and otolaryngologists is essential to achieve etiological precision. Management strategies depend on the underlying cause and include medical or surgical treatment for reversible conditions, intranasal corticosteroids for inflammatory diseases, and olfactory training for post-infectious or congenital forms. Regular follow-up with objective testing and family education supports recovery and long-term adaptation. Despite the scarcity of pediatric-specific evidence, this review highlights the need for awareness, early diagnosis, and individualized management of OD in children, proposing a practical diagnostic and therapeutic framework to guide clinical decision-making in everyday ENT practice. A structured search strategy was applied to summarize the currently available evidence and highlight practical implications for clinical care.

## Introduction

Olfactory perception plays a critical role not only in nutrition but also in social interaction and cognitive development, influencing behaviors across all stages of life, including the establishment of early parent-child bonds. Although olfactory disorders (OD) are relatively common in the general population, with an estimated prevalence of around 22% and increasing with age (1, 2), they are considered rare in children; only approximately 2% of patients attending specialized olfactory clinics are under 18 years old (3, 4). Accurate prevalence estimates in the pediatric population remain challenging due to the limited literature and underdiagnosis.

Importantly, the psychological and behavioral consequences of olfactory loss in children are often underestimated. The sense of smell plays a fundamental role in emotional and social development, influencing memory, bonding, and perception of environmental cues. Children with OD may experience reduced appetite, less enjoyment of food-related activities, and difficulties in forming scent-based emotional associations, which can negatively affect quality of life and psychosocial well-being. Moreover, OD in children may be underdiagnosed or underestimated, as they often fail to report any impairment—possibly because they do not recognize the deficit. This is particularly evident in congenital forms, such as Kallmann syndrome, in which the diagnosis typically relies on other clinical features, and OD is only suspected and subsequently confirmed at a later stage.

This mini review aims to propose a practical flowchart for the diagnosis and treatment of OD in children. Given the limited and fragmented literature available in this field, a targeted literature search strategy was applied to identify the most relevant pediatric studies and expert recommendations, aiming to synthesize current knowledge and support everyday ear, nose and throat (ENT) clinical practice. The authors outline each step of the diagnostic process, from clinical history and identification of potential causes to physical examination and relevant radiological and specialized evaluations, with the goal of achieving an accurate diagnosis and guiding appropriate management.

## Materials and methods

A targeted literature search was conducted in PubMed and Scopus using the main keywords related to pediatric OD in various combination (e.g., “Olfaction Disorders”, “Anosmia”, “Hyposmia”, “Child”, “Pediatrics”). Articles published in English in the last 15 years were considered, with a focus on evidence applicable to clinical ENT practice. Additional references were identified through reference list screening.

### Medical history

**Figure 1:**
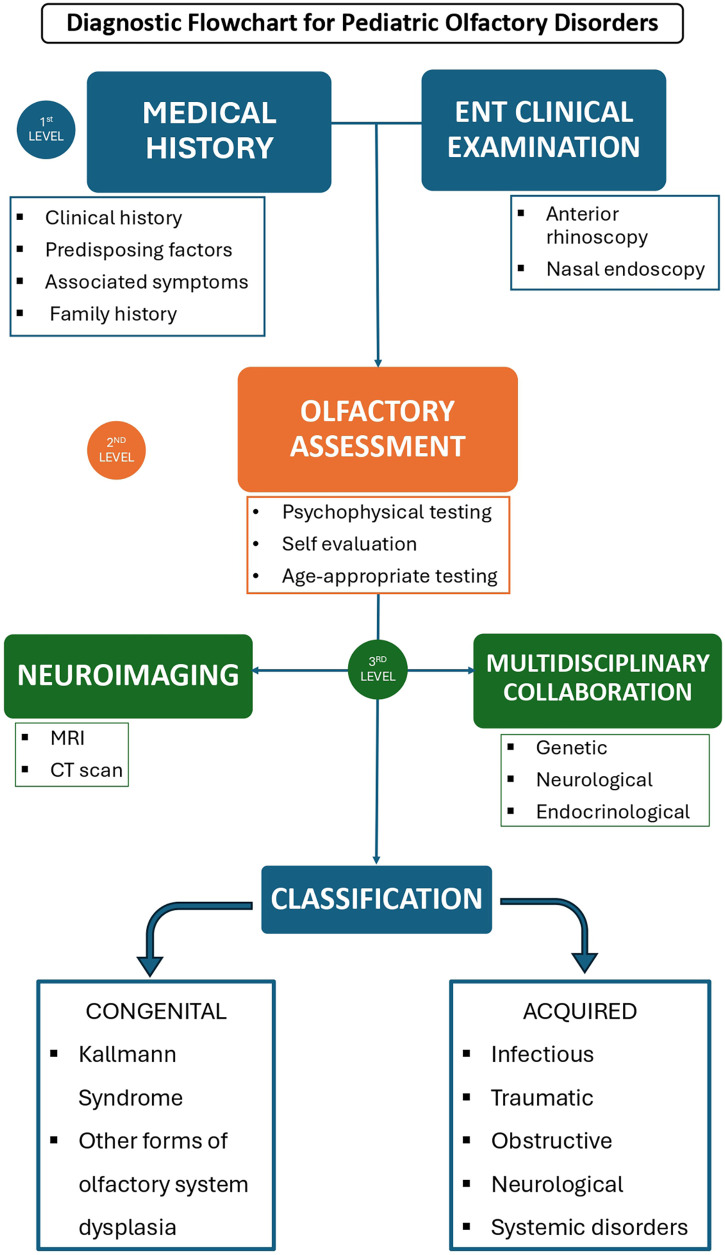
Diagnostic flowchart for pediatric olfactory disorders.

A systematic and structured assessment is essential for the evaluation of olfactory dysfunction in children. The first step involves a comprehensive clinical history, focusing on the onset, duration, and progression of olfactory loss. Establishing whether the deficit is congenital or acquired, transient or persistent, stable, or progressive, provides critical information for narrowing the differential diagnosis and guiding further investigations (Figure 1).

A wide range of predisposing factors should be considered. Among congenital conditions, clinicians should be aware of syndromic disorders such as Kallmann syndrome, characterized by hypogonadotropic hypogonadism and anosmia, and CHARGE syndrome, in which primary olfactory bulb defects may contribute to dysfunction (5). Additional congenital causes include Turner syndrome, Bardet-Biedl syndrome, where impaired ciliary function disrupts olfactory sensory neurons (6), and cleft palate, where anatomical, genetic, or neurological factors can compromise olfaction (7).

Acquired causes are also highly relevant in pediatric populations and include post-viral infections, particularly Severe Acute Respiratory Syndrome Coronavirus 2 (SARS-CoV-2), which may induce temporary or persistent olfactory loss, head trauma or intracranial masses (benign or malignant) affecting olfactory pathways. Structural nasal conditions such as adenoid hypertrophy should be acknowledged, with evidence suggesting recovery of olfactory function following adenoidectomy (8, 9). Other acquired factors include nasal polyps, especially in children with cystic fibrosis, and allergic rhinitis, where disease severity may correlate with olfactory impairment (10). Iatrogenic causes, such as previous endoscopic endonasal surgery, can induce hyposmia, which may be transient or persistent (11). Finally, systemic diseases (e.g., hypothyroidism, other hormonal disorders) and neurological diseases, including autism spectrum disorders (ASD) (12), as well as exposure to medications or environmental toxins, should also be evaluated.

Generally, congenital and post-traumatic causes are more prevalent in younger children. As age increases, these etiologies are progressively replaced by post-infectious and idiopathic causes (3).

In addition to identifying predisposing factors, clinicians should document associated symptoms such as nasal congestion or obstruction, and any neurological deficits, which may indicate central involvement of the olfactory pathways. A detailed family history is equally important, as hereditary olfactory dysfunction—including congenital anosmia or syndromic forms—can provide important diagnostic clues and suggest a genetic predisposition.

### ENT clinical examination

The ENT clinical examination should be comprehensive. First, any craniofacial deformities or anomalies must be documented and considered. Examination includes inspection of the oral cavity and anterior rhinoscopy, which allows primary assessment of inferior turbinate hypertrophy, the presence of nasal secretions (e.g., serous or mucous), and visible nasal polyps. For a more detailed evaluation, both flexible and rigid nasal endoscopy can be performed. Flexible endoscopy enables exploration of all meatuses (inferior, middle, and superior), assessment of anatomical anomalies of the middle turbinate, and detection of obstructions in the nasopharynx, such as adenoid hypertrophy or choanal atresia (13). The olfactory region can also be examined for potential obstruction. Nasal polyps can be classified using standardized scoring systems, the most commonly applied being the Nasal Polyp Score (14). Finally, in patients who have undergone prior surgery, endoscopy allows assessment of surgical outcomes, including synechiae, anatomical changes, and recurrence of pathology.

### Olfactory assessment

The next step in the diagnostic process involves structured olfactory testing, integrating psychophysical, subjective, and age-appropriate assessments to accurately characterize olfactory function in children. The absence of standardized diagnostic protocols for children represents a significant barrier to the early identification and management of OD. This lack of clear guidelines limits opportunities for effective screening and contributes to delayed diagnosis, potentially compromising timely treatment and recovery (15). A concise overview of validated olfactory assessment tools currently available for pediatric populations is provided in Table 1.

**Table 1:** Validated psychophysical olfactory assessment tools in pediatric populations.

Test	Age range	Components	Strengths	Limitations
Sniffin’ sticks test (SSIT) (16, 18, 19)	≥6 years	Threshold, discrimination, identification (TDI score)	Widely validated; broad clinical use; normative data available	Requires attention and familiarity with odors; test can be lengthy
Universal sniff (U-Sniff) (21)	≥6 years	12-odor identification with picture-based answers	International adaptation; child-friendly format	Only identification component; cultural validation still expanding
NIH toolbox odor identification test (NIH-TB) (22)	≥5 years	6-odor identification with distractors	Designed for children; quick administration	Limited odor set; less sensitive to mild dysfunction
Pediatric Barcelona olfactory test-6 (pBOT-6) (23)	≥6 years	Threshold (Phenyl Ethyl Alcohol-PEA) + identification	Combined approach in a brief format	Validated only in Spanish cohort thus far
Pediatric smell wheel (24,25)	≥4 years	Identification using rotating wheel with images	Engaging; suitable for early developmental ages	Limited availability; requires cultural adaptation
UPSIT (child versions available in some settings) (17)	≥6–8 years	Identification only	Highly standardized; large adult database	Not specifically designed for children; cultural familiarity required
Retronasal olfaction test (solution-based tasting — experimental) (27)	Variable	Hedonic perception of odorized solutions	May detect smell impairment not seen orthonasally	Still experimental; not routinely available

Psychophysical assessment represents a cornerstone in the evaluation of pediatric OD and enables comparisons across age groups and clinical conditions. Among the standardized and validated tools, the Sniffin’ Sticks Test (SSIT) (16), provides a composite Threshold–Discrimination–Identification (TDI) score, while the University of Pennsylvania Smell Identification Test (UPSIT) (17), is an identification-only test that quantifies the ability to recognize specific odors from multiple-choice options.

The SSIT remains one of the most used and validated tools in the world for both adults and children. Because odor identification is heavily influenced by familiarity and cultural background, these tests have been translated and culturally adapted into multiple languages to preserve diagnostic reliability. The Pediatric Sniffin’ Stick test has shown good feasibility and reliability in children over 6 years of age, although normative data remain limited and cross-cultural validation is ongoing (18, 19).

Recent literature (20) recommends several tests for valid and reliable olfactory assessment in children. These include the Universal Sniff Odor Identification Test (U-Sniff) which consist of 12 odor pens with picture-based multiple-choice identification (21); the National Institutes of Health Toolbox (NIH-TB) which uses six familiar odors for children, requiring identification among distractors and providing valid, age-appropriate olfactory assessment (22); the Pediatric Barcelona Olfactory Test-6 (pBOT-6) which combines six odorants for forced-choice identification and a six-dilution phenyl ethyl alcohol series for threshold testing (23) and the Pediatric Smell Wheel, designed with simplified odor sets and visual cues to enhance engagement and comprehension while reducing fatigue in early developmental stages (24, 25).

In addition to psychophysical testing, subjective assessments play a complementary role. Self-reported measures, pediatric-adapted questionnaires, and parental observations can capture the child's perception of smell-related difficulties, behavioral changes, and quality-of-life impact—especially when formal testing is limited by age, attention span, or comprehension.

Despite these advances, olfactory testing in children remains methodologically challenging. Younger patients may display reduced motivation, difficulty understanding instructions, or unfamiliarity with specific odors, potentially leading to variable results. Beyond psychophysical methods, electrophysiological techniques—such as olfactory event-related potentials (OERPs)—offer objective insights into olfactory pathway function, but pediatric applications remain limited (26).

These diagnostic tools provide a standardized framework for evaluating olfactory capacity in children. Moreover, retronasal olfactory tests may offer additional diagnostic insights, as adenoid hypertrophy—a common condition in childhood—appears to impair retronasal olfaction more prominently than orthonasal function (27). In clinical practice, children undergoing adenoidectomy are often reported to eat more varied foods than before surgery, which clinicians attribute to an improvement in retronasal olfaction. However, the test proposed by Colbert et al. (27), which involves sampling solutions containing either taste or odor compounds, should be introduced into clinical practice to objectively assess retronasal olfaction. Comparing test results before and after surgery could demonstrate the benefit of adenoidectomy in improving olfactory function.

### Instrumental examinations

Neuroimaging plays a key role in the diagnostic evaluation of OD in selected pediatric cases. In recent years, the use of magnetic resonance imaging (MRI) has significantly advanced the radiological assessment of the olfactory system. MRI allows high-resolution visualization of the olfactory bulbs (OB), olfactory tracts (OT), and adjacent forebrain structures that may be implicated in both congenital and acquired forms of olfactory dysfunction. Moreover, functional MRI (fMRI) contributes to the identification of structural and functional abnormalities, including hypoplasia or aplasia of the OB, providing valuable insights into the neuroanatomical correlates of olfactory loss.

Several studies have demonstrated a strong correlation between OB volume and olfactory function, with patients affected by OD showing significantly smaller bulbs compared to healthy controls (28–31). MRI also facilitates the detection and differentiation of intracranial masses or space-occupying lesions that may contribute to secondary olfactory impairment. One of the major advantages of MRI in pediatric populations is the absence of ionizing radiation, making it particularly suitable for repeated or follow-up imaging in children.

Conversely, a computed tomography (CT) scan remains the preferred imaging modality when bony or sinonasal abnormalities are suspected. CT provides excellent definition of the bony structures of the nasal cavity and paranasal sinuses, enabling accurate evaluation of traumatic injuries, anatomical deformities, and iatrogenic changes. Therefore, MRI and CT should be regarded as complementary techniques, each providing distinct but synergistic diagnostic information in the assessment of pediatric olfactory disorders.

### Multidisciplinary collaboration

Beyond radiological imaging, neurological evaluation can help identify associated central nervous system abnormalities or neurodevelopmental disorders that may contribute to OD. When a systemic or endocrine disease is suspected—such as hormonal dysfunction or metabolic disorders—collaboration with an endocrinologist is recommended to ensure a comprehensive diagnostic framework. In cases of congenital olfactory disorders, genetic assessment is fundamental to confirm the diagnosis and guide appropriate counseling for patients and families. Finally, close cooperation with maxillofacial surgeons is often necessary for the evaluation and correction of craniofacial or bony anomalies that may underline or exacerbate olfactory impairment.

The pediatrician plays a central role in this multidisciplinary network, acting as the primary coordinator who integrates clinical information, guides referrals, and ensures continuity of care among the various specialists involved. Integrating these disciplines ensures a holistic diagnostic framework, improving etiological accuracy and guiding personalized treatment strategies. Therefore, a multidisciplinary approach is essential for the accurate assessment and effective management of OD in children.

### Management and treatment

**Figure 2:**
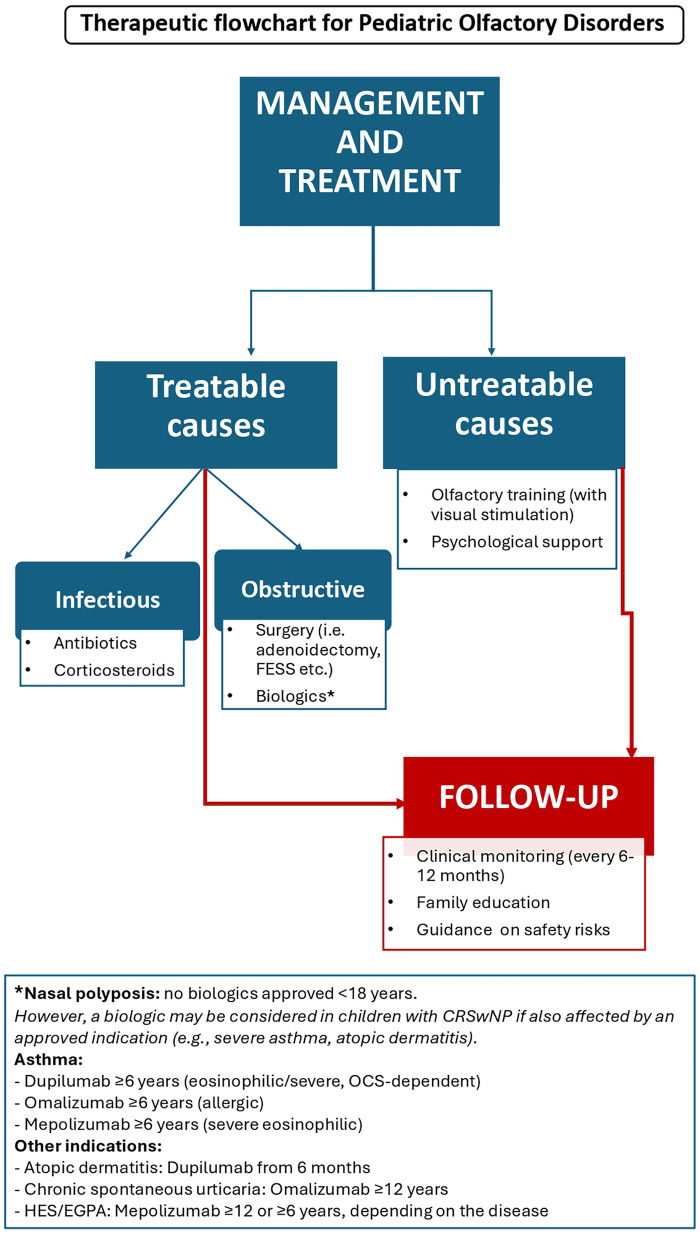
Therapeutic flowchart for pediatric olfactory disorders.

The management of pediatric OD depends on the underlying etiology and can be broadly categorized into treatable and non-treatable conditions. Identifying the root cause is essential to guide targeted therapy and optimize functional recovery (Figure 2).

Among treatable causes, obstructive and structural disorders are particularly relevant. Surgical interventions can restore nasal patency and improve olfactory function. Common procedures include adenoidectomy for adenoid hypertrophy, Functional Endoscopic Sinus Surgery (FESS) for nasal polyps, and turbinoplasty for turbinate hypertrophy (32). Surgical management may also be indicated for nasal or intracranial masses, with the approach tailored according to benign or malignant pathology.

Infectious and inflammatory conditions are typically managed with medical therapy (e.g., antibiotics, corticosteroids), both in acute and chronic rhinosinusitis. However, in selected chronic cases, surgical intervention may be necessary. Intranasal corticosteroids have demonstrated efficacy not only in sinonasal diseases such as nasal polyposis, but also in post-viral inflammatory anosmia, reducing local inflammation and improving olfactory function (33). According to some published protocols, the treatment may include budesonide irrigations once daily by adding 0.5 mg/2.0 mL of budesonide into a 200 mL saline sinus irrigation bottle (34). In recent years, biologic therapies have expanded treatment options for related conditions. Although biologics are not formally approved for pediatric chronic rhinosinusitis with nasal polyps (CRSwNP), they may be considered in children who meet criteria for other approved indications, such as severe asthma, atopic dermatitis, or eosinophilic disorders (35–37).

In case of untreatable diseases, including congenital syndromes (e.g., Kallmann syndrome, olfactory bulb hypoplasia/aplasia), management focuses on supportive care, olfactory training, and addressing psychosocial consequences. Olfactory training (OT), consisting of daily exposure to a set of specific odors, has been shown to stimulate functional recovery, particularly in post-infectious or post-traumatic OD (38). In pediatric settings, children are typically encouraged to practice daily with a limited set of familiar scents, chosen to maintain motivation and adherence. The selection of odors can be individualized rather than standardized, and training is often supported by parental supervision or the use of a simple diary to document adherence (34).

Ultimately, effective management requires a multimodal approach, integrating surgical, medical, and rehabilitative strategies while considering the child's overall development and quality of life. Tailoring therapy to the underlying cause and providing supportive interventions ensures not only functional recovery but also addresses the emotional and social impact of olfactory loss.

### Follow-up

Once the underlying causes have been identified and appropriate treatment has been initiated, follow-up is essential to monitor the course and progression of pediatric OD. Clinical monitoring should include repeat olfactory testing and comprehensive ENT evaluations at regular intervals, typically every 6–12 months. These assessments provide psychophysical information on olfactory function, including threshold, discrimination, and identification performance, and enable the early detection of deterioration or improvement, particularly in cases of post-infectious, post-traumatic, or surgically treated OD.

Regular follow-up also allows for the evaluation of associated nasal or systemic conditions, including recurrent sinus infections, allergic rhinitis, or structural changes following surgery. In children receiving medical therapy, such as intranasal corticosteroids or biologics, repeated assessments can help determine treatment efficacy and guide adjustments in dosage or therapeutic approach. Similarly, in patients undergoing olfactory training, periodic evaluations provide feedback on adherence, performance, and potential benefits, supporting optimization of the rehabilitation program.

Family education represents an equally important aspect of follow-up care. Children with OD are at increased risk for environmental hazards, including gas leaks, smoke, spoiled food, or fire. Parents and caregivers should be counseled on safety measures and strategies to mitigate these risks. Additionally, guidance on dietary adaptations, olfactory cues for daily activities, and psychosocial support can improve quality of life and foster independence, particularly in younger children.

A structured follow-up protocol, combining objective olfactory testing, clinical reassessment, and family education, ensures a holistic approach to pediatric OD. It allows clinicians to track the course of the disorder, tailor interventions over time, and provide families with the knowledge and resources necessary to manage the functional and safety challenges associated with olfactory loss. By integrating clinical, rehabilitative, and educational strategies, follow-up care supports both recovery and long-term adaptation, addressing the multifaceted impact of OD in childhood.

### Limitations

This review has several limitations. Being narrative in nature, it does not follow a systematic review methodology and may therefore be subject to selection bias. The available literature on pediatric OD is sparse and heterogeneous, often based on small cohorts or extrapolated from adult data. Diagnostic and therapeutic recommendations are therefore largely based on expert opinion rather than high-level evidence. Moreover, psychophysical tests validated in adults are only partially adapted for children, limiting comparability across studies. Finally, cultural and linguistic differences further challenge the interpretation of olfactory test results and the generalizability of findings.

Despite these limitations, the proposed approach provides a pragmatic and multidisciplinary framework that may assist clinicians in structuring diagnostic reasoning, improving etiological accuracy, and raising awareness of the significance of olfactory loss in children. Future multicenter and longitudinal studies are needed to clarify the pathophysiological links between allergic airway inflammation and smell impairment, to validate pediatric-specific diagnostic tools, and to assess the efficacy of medical and rehabilitative interventions.

## Discussion

This review summarizes current evidence and proposes a practical algorithm to guide the evaluation and management of OD in children which remains underdiagnosed and under-investigated. The paucity of standardized diagnostic pathways, the limited availability of age-appropriate testing tools, and the absence of normative data across developmental stages represent major barriers to accurate assessment.

Recently literature has begun to address these challenges. The scoping review by Payandeh et al. (39) identified more than forty potential etiologies of pediatric OD and highlighted the predominance of sensorineural mechanisms in the pediatric population. Their work emphasized the urgent need for validated age-appropriate olfactory tests to improve comparability and early detection. In fact, a major challenge remains the limited availability of age-stratified normative data, particularly in preschool and early school-age children, which substantially affects the interpretation and comparability of psychophysical testing results. Likewise, Gellrich and colleagues (15) proposed a stepwise diagnostic pathway that integrates psychophysical assessment, imaging, and multidisciplinary collaboration, confirming the need for structured clinical reasoning in ENT and allergy practice.

Our proposed flowchart aligns with this approach and integrates clinical, instrumental, and multidisciplinary steps into a structured decision-making pathway that can be applied in the ENT outpatient setting. It emphasizes the importance of detailed medical history, comprehensive endoscopic examination, and tailored psychophysical testing to differentiate congenital from acquired causes. Moreover, it highlights the complementary role of neuroimaging and the necessity of involving multiple specialists, including neurologists, endocrinologists, geneticists, and pediatricians, to achieve etiological precision.

From a clinical standpoint, several aspects deserve attention. First, congenital and syndromic anosmia, although rare, should always be considered in children presenting with early-onset or lifelong OD, especially when associated with systemic or developmental anomalies. Second, the increasing prevalence of post-viral and post-traumatic olfactory loss underscores the need for early recognition and follow-up to monitor spontaneous recovery or response to interventions. Third, the psychosocial impact of olfactory impairment in childhood—often underestimated—should be integrated into the clinical evaluation, as it may affect nutrition, emotional development, and safety awareness.

Also, the interplay between inflammatory airway diseases and olfactory impairment in allergic rhinitis, chronic rhinosinusitis with or without nasal polyps, and adenoid hypertrophy deserves particular attention. Standardized assessment of retronasal olfaction is still in its infancy in children, despite its potential value in these conditions, where early identification and treatment may contribute not only to symptom control but also to olfactory recovery. The proposed flowchart integrates these aspects, underlining the need for an accurate differentiation between congenital, inflammatory, and obstructive causes.

Emerging therapeutic strategies such as olfactory training and biologic therapies hold promise for selected pediatric cases, but evidence remains scarce. Standardization of olfactory assessment tools for different age groups and cultural contexts is urgently needed to enable consistent diagnosis, facilitate longitudinal monitoring, and support interventional studies. Furthermore, evidence on treatment efficacy remains largely extrapolated from adult studies, underscoring the need for pediatric-specific clinical trials and longitudinal research. Multicenter collaborations could help establish normative data and identify reliable biomarkers for prognosis and treatment response.

Future systematic reviews and meta-analyses will be crucial as higher-quality pediatric data become available, enabling a more robust quantitative synthesis and stronger evidence-based recommendations.
